# Supplementary material for: Spatial Pattern Characteristics and Factors for the Present Status of Rural Settlements in the Lijiang River Basin Based on ArcGIS
Source: Int J Environ Res Public Health. 2023 Feb 25;20(5):4124. doi: 10.3390/ijerph20054124 (PMC10001701; doi:10.3390/ijerph20054124)
Supplement: Supplementary file 1 [file ijerph-20-04124-s001.zip › ijerph-2152077-supplementary.pdf]

Supplemental Table S1. The number and area of rural settlements with different elevations and slopes

Index	Number of rural settlements	Percentage (%)	Area (hm^2)	Percentage (%)	Average area (hm^2)
Elevation					
0-100	39	2.7%	466.09	4.2%	11.95
100-200	1214	83.0%	9588.72	86.6%	7.90
200-300	170	11.6%	796.69	7.2%	4.69
300-400	21	1.4%	84.27	0.8%	4.01
>400	19	1.3%	132.18	1.2%	6.96
Slope					
0° - 0.5°	123	8.4%	988.35	8.9%	8.04
> 0.5° - 2°	229	15.7%	1867.32	16.9%	8.15
> 2°- 5°	670	45.8%	5149.19	46.5%	7.69
> 5°- 15°	309	21.1%	2339.59	21.1%	7.57
> 15°- 35°	113	7.7%	609.97	5.5%	5.40
> 35°- 55°	15	1.0%	97.16	0.9%	6.48
> 55°- 90°	4	0.3%	16.37	0.1%	4.09
Distance					
0-0.5	767	52.4%	5344.17	48.2%	6.97
0.5-1	350	23.9%	2905.42	26.2%	8.30
1-1.5	173	11.8%	1703.18	15.4%	9.84
1.5-2	105	7.2%	653.46	5.9%	6.22
2-2.5	48	3.3%	355.51	3.2%	7.41
2.5-3	15	1.0%	94.04	0.8%	6.27
>3	5	0.3%	21.02	0.2%	4.20
