# Supplementary figures and images for: Spatial Pattern Characteristics and Factors for the Present Status of Rural Settlements in the Lijiang River Basin Based on ArcGIS
Source: Int J Environ Res Public Health. 2023 Feb 25;20(5):4124. doi: 10.3390/ijerph20054124 (PMC10001701; doi:10.3390/ijerph20054124)

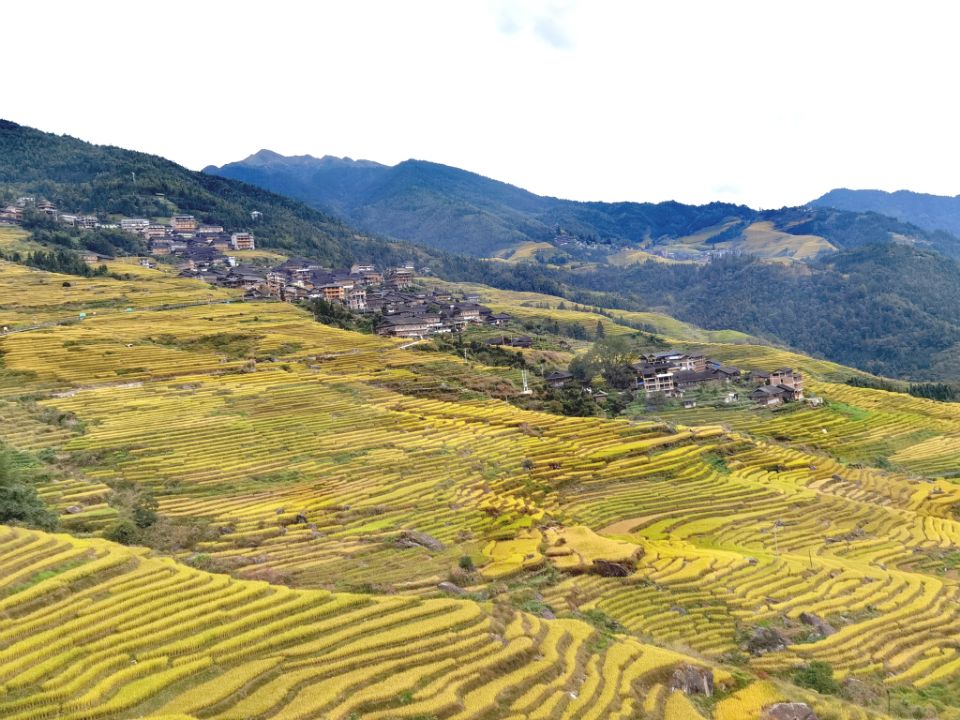

Supplement: Supplementary file 1 [file ijerph-20-04124-s001.zip › Supplemental Figure S1.jpg]

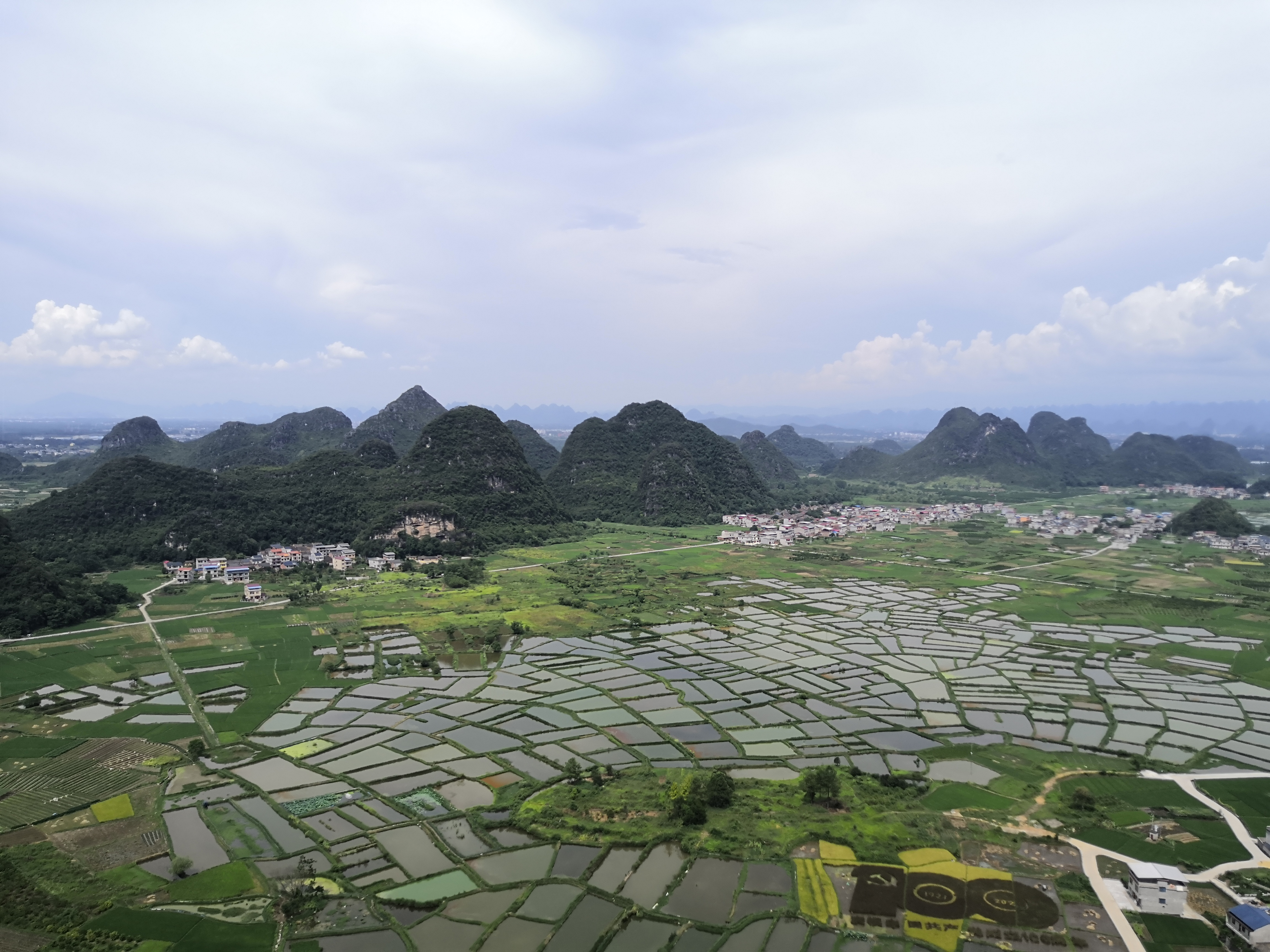

Supplement: Supplementary file 1 [file ijerph-20-04124-s001.zip › Supplemental Figure S2.jpg]

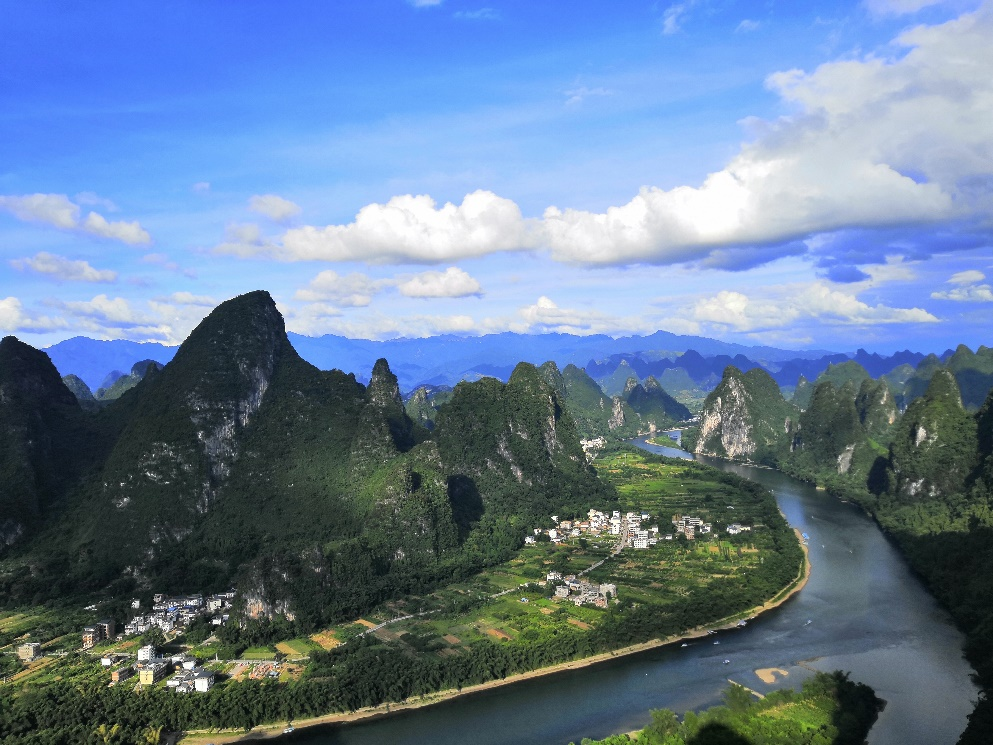

Supplement: Supplementary file 1 [file ijerph-20-04124-s001.zip › Supplemental Figure S3.jpg]

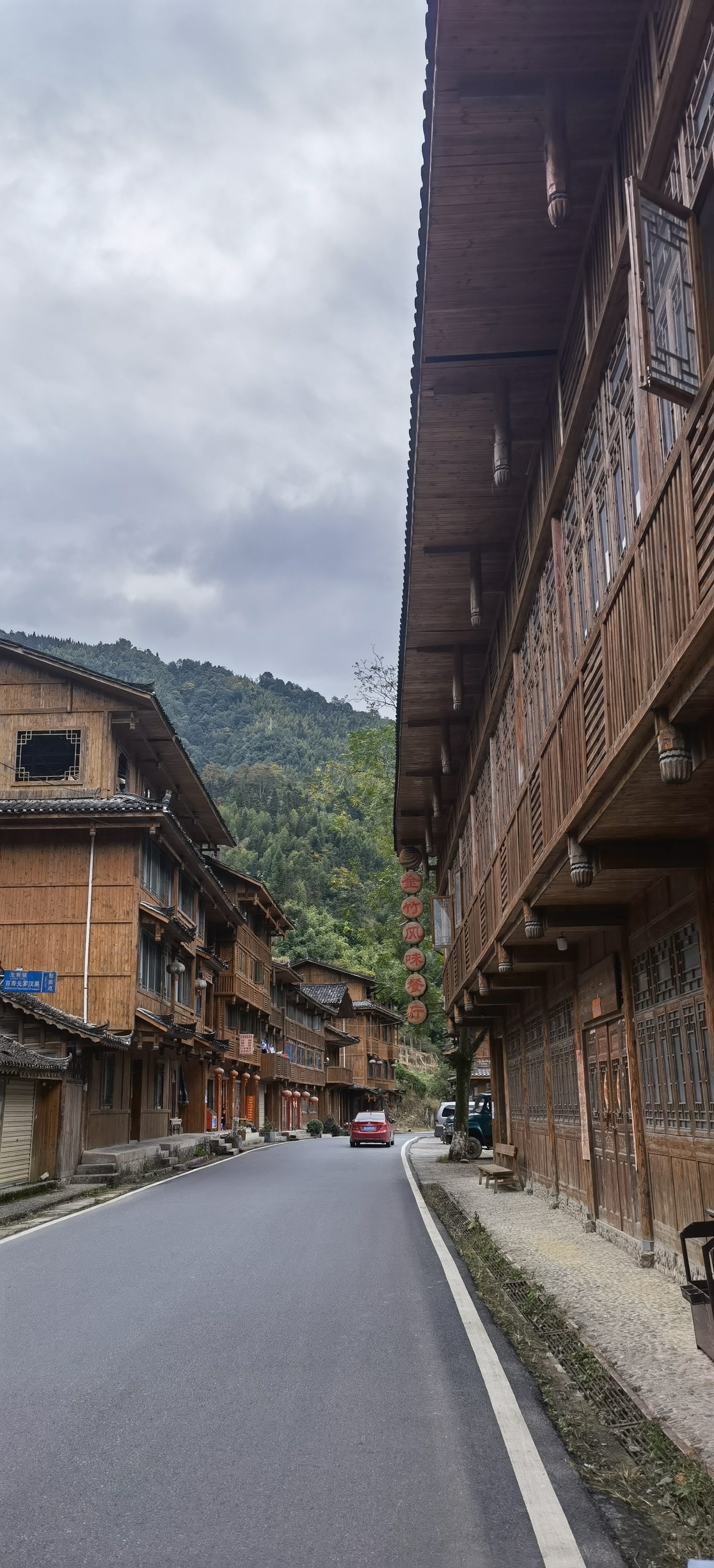

Supplement: Supplementary file 1 [file ijerph-20-04124-s001.zip › Supplemental Figure S4.jpg]
